# 1207. Epidemiology and Timing of Infectious Complications from Battlefield-Related Burn Injuries

**DOI:** 10.1093/ofid/ofac492.1040

**Published:** 2022-12-15

**Authors:** Matthew Geringer, Laveta Stewart, Faraz Shaikh, Leigh Carson, Dan Lu, Leopoldo C Cancio, Jennifer M Gurney, David R Tribble, John L Kiley

**Affiliations:** Brooke Army Medical Center / San Antonio Uniformed Services Health Education Consortium, San Antonio, Texas; Infectious Disease Clinical Research Program, Bethesda, Maryland; Infectious Disease Clinical Research Program, Bethesda, Maryland; Infectious Disease Clinical Research Program, Bethesda, Maryland; HJF, Rockville, Maryland; US Army Institute of Surgical Research, San Antonio, Texas; US Army Institute of surgical research/joint trauma system, San Antonio, Texas; Uniformed Services University of the Health Sciences, Bethesda, Maryland; BAMC, San Antonio, Texas

## Abstract

**Background:**

Thermal injury alters the host response, making burn patients more susceptible to infections. In fact, infections represent the most frequent complication and cause of mortality in burn patients. We describe the epidemiology, clinical characteristics, timing, and outcomes of infections among wounded military personnel with burns.

**Methods:**

Data were collected through the Trauma Infectious Disease Outcomes Study, an observational study of US service members injured in Iraq and Afghanistan (6/09-12/14). Patients who sustained ≥1 burn injury and were admitted to the Burn Center at Brooke Army Medical Center were included in the analysis. Infections were defined using standardized criteria. For patients with multiple infections, only the initial infection was assessed.

**Results:**

Among 144 burn patients, 99% were males and 62% had combat-related burns with a median total body surface area (TBSA) of 6% (IQR 3-14%) thermally injured. Infections were diagnosed in 26 (18%) patients with pneumonia being the predominant initial syndrome (N=16, 62%), followed by skin and soft-tissue infections (N=6, 23%), bloodstream infections (N=3, 12%), and intra-abdominal infections (N=1, 4%). Median number of days to each of these initial infecting syndromes were 4 (IQR 3-5), 7 (IQR 4-12), 7 (IQR 6-7), and 17 (IQR 17-17) days, respectively. Patients with infections were more severely injured with greater TBSA (median 31 vs 5) and Baux scores (median 59 vs 29), and were more likely to have combat trauma, inhalation injury, require mechanical ventilation, and have longer time to definitive grafting (Table 1). Microbiology of initial infections varied with 35% of patients having polymicrobial infections (Table 2). Gram-negative organisms were recovered from 20 (77%) patients, of whom 20% had a multidrug-resistant Gram-negative. Gram-positive organisms and fungi were identified in 42% and 8% of patients, respectively.

**Figure:**
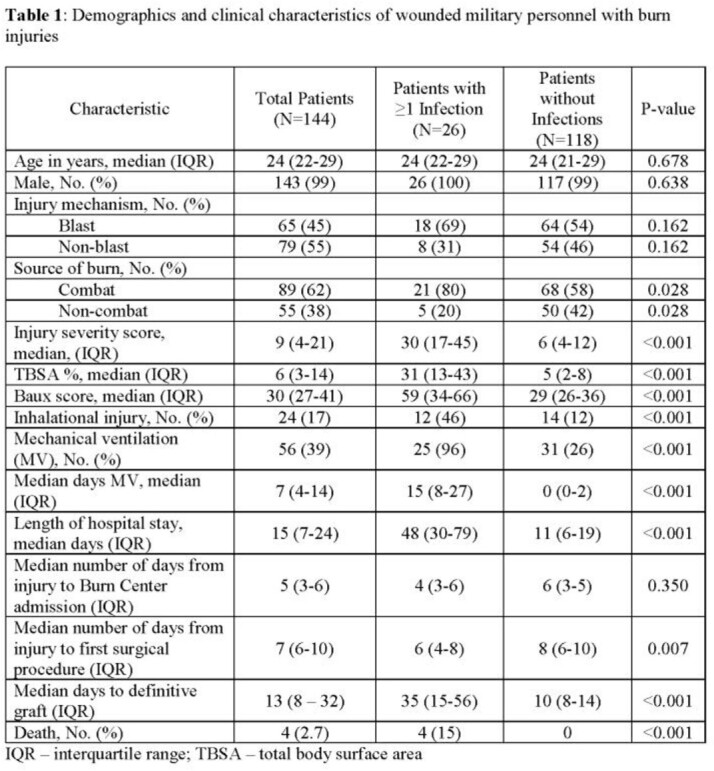

**Figure:**
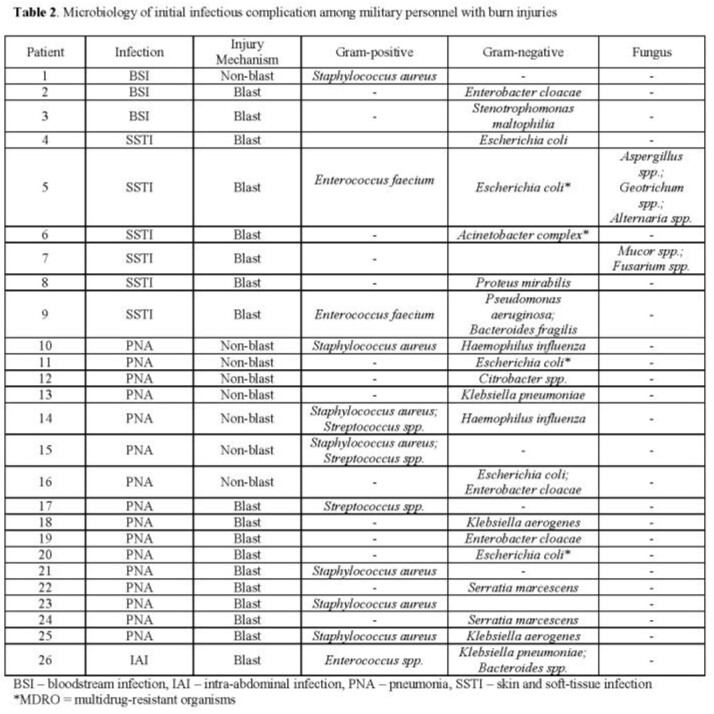

**Conclusion:**

Improved understanding of risk factors and the timing of infections in this unique population is critical for effective management. Patients with infections were more severely injured, had higher rates of inhalational injury, and longer days to definitive grafting. Initial infections were more commonly pneumonia.

**Disclosures:**

**David R. Tribble, DrPH**, AstraZeneca: The HJF, in support of the USU IDCRP, was funded to conduct or augment unrelated Phase III Mab and vaccine trials as part of US Govt. COVID19 response.

